# Psychological interventions for early-phase schizophrenia: protocol for a systematic review and network meta-analysis

**DOI:** 10.12688/f1000research.152191.1

**Published:** 2024-06-17

**Authors:** Lena Feber, Georgia Salanti, Mathias Harrer, Nurul Husna Salahuddin, Wulf-Peter Hansen, Josef Priller, Irene Bighelli, Stefan Leucht

**Affiliations:** 1 Technical University of Munich, TUM School of Medicine and Health, Klinikum rechts der Isar, Department of Psychiatry and Psychotherapy, Munich, Germany, Munich, Germany; 2 Institute of Social and Preventive Medicine (ISPM), University of Bern, Bern, Switzerland, Bern, Switzerland; 3 BASTA—Bündnis für psychisch erkrankte Menschen, Munich, Germany, Munich, Germany; 4 German Center for Mental Health (DZPG), Munich, Germany, Munich, Germany; 5 Neuropsychiatry and Laboratory of Molecular Psychiatry, Charité – Universitätsmedizin Berlin, and DZNE, Berlin, Germany, Berlin, Germany; 6 University of Edinburgh and UK DRI, Edinburgh, UK, Edinburgh, UK

**Keywords:** Schizophrenia, Psychological Interventions, Early Phase, First Episode, Network meta-analysis

## Abstract

**Introduction:**

Treating the early phase of schizophrenia is crucial for preventing further episodes and improving quality of life, functioning, and social inclusion. Pharmacotherapies are first-line treatments, but have limitations. There is consensus on the need for non-pharmacological interventions for individuals in the early phase of schizophrenia. Several psychological interventions have shown promising effects; however, their comparative effectiveness remains largely unknown. To address this issue, a network meta-analysis will be performed. We aim to develop a hierarchy of existing psychological treatments concerning their efficacy and tolerability, which will inform treatment guidelines.

**Protocol:**

Randomized controlled trials (RCTs) investigating psychological interventions for first-episode psychosis, first-episode schizophrenia, or early phase schizophrenia will be included. The primary outcome will be overall schizophrenia symptoms (measured up to 6 and 12 months, and at the longest follow-up) and relapse as a co-primary outcome. Secondary outcomes are premature discontinuation; change in positive, negative, and depressive symptoms of schizophrenia; response; quality of life; overall functioning; satisfaction with care; adherence; adverse events; and mortality. The study selection and data extraction are performed by two independent reviewers. We will assess the risk of bias of each study using the Cochrane Risk of Bias tool 2 and evaluate the confidence in the results using Confidence in Network Meta-Analysis (CINeMA). Subgroup and sensitivity analyses will be conducted to explore heterogeneity and assess the robustness of our findings.

**Discussion:**

This systematic review and network meta-analysis aims to compare multiple existing psychological interventions, establishing which are best for symptom reduction, relapse prevention, and other important outcomes in early phase schizophrenia. Our results may provide practical guidance concerning the most effective psychological intervention to reduce symptom severity and the societal burden associated with the disorder.

**List of abbreviations**
BPRS: Brief Psychiatric Rating Scale
CGI: Clinical Global Impression
CI: Confidence interval
CINeMA: Confidence in Network Meta-Analysis
GAF: Global Assessment of Functioning
GRADE: Grading of Recommendations, Assessment, Development and Evaluation
IQWIG: Institut für Qualität und Wirtschaftlichkeit im Gesundheitswesen
LOCF: Last observation carried forward
MMRM: Mixed-models of repeated measurement
NMA: Network meta-analysis
OR: Odds ratio
PANSS: Positive and Negative Syndrome Scale
PPS: Psychosocial Performance Scale
PRISMA: Preferred Reporting Items of Systematic Reviews and Meta-Analyses for Systematic Review Protocols
PROSPERO: International Prospective Register of Systematic Reviews
QOLS: Quality of Life Scale
RCT: Randomized controlled trial
RoB: Risk of Bias
SD: Standard deviation
SE: Standard error
SIDE: Separating indirect evidence from direct evidence
SMD: Standardized mean difference
SUCRA: Surface under the cumulative ranking curve
TAU: Treatment as usual

## Introduction

Schizophrenia is a debilitating and often life-long disorder that ranks among the 20 top causes of disability. ^1^ The typical age of onset is in the early 20s, ^2^ often resulting in a dramatic reduction in patients’ quality of life, social functioning, educational attainment, and socioeconomic status. There is an agreement on the importance of treating this critical phase to prevent further episodes. ^3^^,^^4^ Different definitions have been used to refer to the early course of the illness, and the term ‘first episode’ is often used in a broader sense. ^5^ Research focuses on the early phases, including the second or third episode, usually with a time from onset criterion. ^5^

Pharmacological interventions are a first-line treatment for schizophrenia but have several limitations, including limited response, high incidence of disabling side effects, and poor adherence to treatment. ^6^^,^^7^ Antipsychotics can also be problematic for patients with medical comorbidities, tolerability problems, or pregnancy. Overall, 24% of patients with a first episode of psychosis did not respond when treated with antipsychotics, ^8^ and 26% experienced relapse. ^9^ Furthermore, few antipsychotics are licensed for people under 18 years of age and tolerability problems are higher in this subpopulation. ^4^ The resulting burden on patients, relatives, and society as a whole is substantial, with acute symptoms and relapses often leading to costly hospitalizations, job loss, and strained relationships. As a result, there is a strong agreement on the need for non-pharmacological interventions in patients in the early phase of schizophrenia. ^4^ Knowledge of the psychological interventions that are most efficacious in improving symptoms and preventing relapse in this critical population may improve treatment planning and resource allocation in the healthcare sector.

Previously, evidence regarding psychological interventions for early phase schizophrenia has been synthesized in pairwise meta-analyses that examined single psychological interventions, such as CBT and family therapies. The effects were mainly compared to those of usual care (TAU), with promising results on social functioning, ^10^^,^^11^ relapse, ^11^^–^^13^ and psychotic symptoms. ^13^ Our group conducted a Cochrane review investigating the role of CBT for this population compared to TAU. ^14^ Early intervention teams, providing collaborative care including medication, psychotherapy, psychoeducation, and occupational, educational, and employment support, have also shown an effect in reducing dropouts and improving functioning compared to TAU. ^15^ Three network meta-analyses have been conducted on psychological interventions for patients with schizophrenia: one on positive symptoms excluded patients with a first episode ^16^; one was strictly focused on relapse prevention, including only studies where relapse was the primary outcome, and found only a small number of studies on patients in the early phase ^17^; the third only considered overall symptoms as outcome, with searches dating back to 2016. ^18^ Therefore, no up-to-date evidence can be derived from these NMAs in patients in the early phase of schizophrenia. However, a very recent network meta-analysis examined psychological interventions for the treatment of early psychosis and demonstrated positive effects. ^19^ Nevertheless, this analysis was based on a limited number of studies, investigated only a small number of outcomes, and had no special focus on schizophrenia. ^19^

In summary, the current evidence on psychological treatments for early stage schizophrenia is limited to pairwise meta-analyses that investigate single interventions (such as cognitive behavioral therapy and family interventions), as well as to NMAs that either do not focus on the corresponding population or include a very small number of studies or outcomes. Thus, the most efficacious and acceptable psychological treatments for patients with schizophrenia in the early phase of the disorder are currently unclear. This underlines the need for a network meta-analysis to produce a comprehensive synthesis of all randomized evidence, focusing on this particular population.

### Objective

The goal of this systematic review and network meta-analysis is to synthesize the relative efficacy of all available psychological treatments in one analysis. We aim to combine both direct and indirect evidence to create effect estimates, even for treatments that have never been directly compared in a trial. Furthermore, we plan to extend the existing knowledge on the topic by examining multiple endpoints, including symptom severity, quality of life, functioning, and relapse rates.

### Protocol

This systematic review and network meta-analysis protocol followed the Preferred Reporting Items for Systematic Reviews and Meta-Analyses (PRISMA-P) statement extension for network meta-analysis. ^20^ Appendix 1 provides the PRISMA-P checklist. This protocol was registered in PROSPERO (CRD42024514287).

### Eligibility criteria

*Characteristics of studies*

We will include (i) randomized controlled trials comparing (ii) a psychological intervention to (iii) a control group (waitlist, treatment as usual, psychological placebo, or other psychosocial or psychological treatments). Psychological placebo will be defined as any condition aimed at controlling the time spent with a therapist, without delivering any *bona fide* psychotherapeutic content (e.g., recreational therapy, activity groups). We expect that, in the majority of studies, psychological intervention will be provided in addition to standard care, which usually includes antipsychotic medication. If we find studies in which psychological intervention is administered without concomitant pharmacotherapy, we will consider these studies as well.

*Characteristics of participants*

We will consider trials in patients with a recent first-time diagnosis of psychosis, first-episode schizophrenia, or early phase schizophrenia, as per the inclusion criteria of specific studies. In cases of “early-phase schizophrenia” or “recent-onset psychosis”, we will accept a maximum duration of illness of 5 years. We will also include studies conducted in specialized early intervention centers since they target the population in question. We will include studies with participants aged 16 years or older, allowing us to capture the variability in the age of onset of schizophrenia. Studies focusing on participants over the age of 65 years as part of their inclusion criteria or those investigating late-onset psychosis will be excluded. We will exclude studies that only include patients with bipolar mania, bipolar depression, substance-induced disorders, and psychoses of organic origin. We enforce this criterion because, despite unavoidable overlap, our focus is on schizophrenia and schizophrenia-related disorders (schizoaffective disorder and delusional disorder). We will exclude studies in “at risk of psychosis” or “prodrome” populations because such patients do not experience full-blown psychotic symptoms by definition. If a study included participants with other diagnoses, we include it if participants with psychosis (as defined above) comprised at least 50% of the participants. There will be no restrictions on sex, ethnicity, or setting.

*Interventions*

We will consider all psychological interventions that meet the classification criteria proposed by the Cochrane Common Mental Disorder Group (former CCDAN). ^21^ These classification criteria have already been employed in a previous meta-analysis on schizophrenia, in which early phase participants were not included. ^16^ We do not expect that studies and data will be available for all interventions mentioned in the classification, but if they do, they will be considered. Thus, we will consider psychotherapies (e.g., cognitive behavioral therapy, “third-wave” cognitive behavioral therapies, such as acceptance and commitment therapy, mindfulness and metacognitive training, psychodynamic therapy, art therapy, and music therapy), treatments that are often routinely provided in clinical settings, such as supportive therapy and psychoeducation, interventions focused on functioning (e.g., social skills training), and family interventions. Newly developed treatments that are not included in the list will also be considered for inclusion (e.g., virtual reality and *AVATAR* therapy). Organization of care-oriented interventions (e.g., case management, assertive community treatment) and supported employment will not be considered, because they have other aims, and it will not be meaningful to analyze their efficacy in contrast to the ones mentioned above.

### Outcomes

*Primary outcome*

Our primary outcome was the severity of schizophrenia symptoms, measured by rating scales such as the Positive and Negative Syndrome Scale (PANSS), Brief Psychiatric Rating Scale (BPRS), or any other validated scale for the assessment of overall symptoms of schizophrenia. Based on our experience, we expect these scales to be used in almost all the trials. If only results of other rating scales are reported, they will only be used if the instrument has been validated in psychosis and published in a peer-reviewed journal, because non-validated schizophrenia scales tend to exaggerate differences. ^22^

Relapse will be the primary outcome. For patients in the early phase, it is crucial to prevent relapse because the risk of chronicity increases with every episode. If more measures of relapse are reported, we will prioritize measures according to the following order: (i) operationalized criteria, (ii) hospital admissions due to psychopathology, and (iii) clinical judgement.

*Secondary outcomes*

We will also consider a broad collection of outcomes that may be informative for patients, clinicians, and guideline makers if available in the primary studies.

1. Premature discontinuation (“dropout”) due to any reason: All-cause discontinuation (‘dropping out’) due to any reason combines efficacy, tolerability, and other factors and can therefore been considered as a measure of ‘acceptability of treatment’. ^23^ We will also separately report the number of dropouts due to inefficacy of the treatment and worsening of clinical conditions.
2. Change in positive, negative and depressive symptoms of schizophrenia: According to the respective subscales of the PANSS or the “Scales for Assessment of Positive/Negative Symptoms” (SAPS, SANS), the Calgary Depression Scale for Schizophrenia, the Hamilton Depression Rating Scale, the Montgomery-Åsberg Depression Scale, or other validated symptom scales. These are important components of the psychopathology of schizophrenia and contribute considerably to the burden associated with the disease.
3. Response: The number of responders is defined based on validated rating scales. We will extract the percentage of participants who experienced at least a 20% reduction in PANSS or BPRS scores or a minimum improvement on the Clinical Global Impression (CGI) scale. These findings can be considered equivalent and meaningful for patients in the early phases of the disorder. ^24^
4. Quality of Life: The importance of this outcome was highlighted by our patient representatives collaborators and recommended by institutions such as the German “Institut für Qualität und Wirtschaftlichkeit im Gesundheitswesen” (IQWIG). We will accept any published, validated rating scale (e.g. “Heinrichs quality of life scale”; Quality of Life Scale, QOLS).
5. Overall Functioning: Measured by rating scales such as the Global Assessment of Functioning (GAF), Psychosocial Performance Scale (PPS), or any other published rating scale. We will also record the number of participants employed, having a relationship, and living independently as more direct measures of functioning, although we anticipate that this will rarely be reported.
6. Satisfaction with care: We will measure participants’ satisfaction with care using any validated scale. This outcome was also highlighted as relevant by our patient representatives.
7. Adherence: The extent to which psychological treatment can contribute to patients’ compliance with treatment in general will also be considered. We will accept any published rating scale (e.g., “Adherence Therapy Patients Satisfaction Questionnaire,” “Adherence Rating Scale”).
8. Adverse events: We will collect any available information in the included studies about this outcome, using the classification proposed by Linden et al. ^25^: (i) emergence of new symptoms, (ii) deterioration of existing symptoms, (iii) lack of improvement or deterioration of illness, (iv) prolongation of treatment, (v) strains in the patient-therapist relationship, (vi) therapy dependency, (vii) strains or changes in family relations, (viii) strains or changes in work relations, (ix) any change in the life circumstances of the patient, and (x) stigmatization. We also included cases of abuse. Patient noncompliance is already covered as a separate outcome and is not considered here. Suicide attempts and other possible adverse events related to psychological treatment should also be considered.
9. Death: Psychological treatment may reduce overall mortality, particularly suicidality. We examine this outcome separately in terms of (i) death for any reason, (ii) death due to natural causes, and (iii) death due to suicide.

## Methods

### Search strategy

We searched the most important electronic databases for relevant publications: BIOSIS, CINAHL, Cochrane CENTRAL, Dissertation Abstracts, EMBASE, LILACS, MEDLINE, PsycINFO, and Pubmed), including clinical trial websites such as Clinicaltrials.gov and the International Clinical Trials Registry Platform of the World Health Organization ( http://apps.who.int/trialsearch). Additionally, we will search previous reviews, statements of guidelines, and specific relevant journals concerning psychological treatments for schizophrenia to retrieve other studies. We will contact the first authors of the included studies in case of missing information. The search strings are provided in the Supplementary Material (Appendix 2). ^50^

### Identification and selection of studies

Two reviewers from our team will independently screen the search results for general inclusion criteria using the online tool Rayyan. ^26^ Disagreements will be resolved by discussion, and, where doubts remain, we will acquire the full article for further inspection. Once the full articles were obtained, the two reviewers will independently decide whether the studies meet the review criteria. If disagreement cannot be clarified by discussion, we will consult a senior researcher (SL, IB) or seek further information from the authors.

### Data extraction

Two reviewers will independently extract data from all selected trials using specifically developed forms in a Microsoft Access database. The forms will be piloted on a random sample of ten randomized controlled trials (RCTs). Disagreements will be resolved by discussion with a third senior researcher (SL, IB). The authors will be contacted if needed to resolve disagreements. The following data will be extracted from each included study: (i) general study information, (ii) information on methodology, (iii) characteristics of study participants, (iv) characteristics of psychological interventions, and (v) outcome measures.

*Measurement of treatment effect*

For continuous outcomes, we will calculate the standardized mean difference (SMD) as the effect measure, since we expect studies to use different rating scales for the measurement of overall schizophrenia symptom severity. We will prefer results obtained with imputation methods to handle missing data over complete data; results from mixed models of repeated measurement (MMRM) or multiple imputations will be preferred over last observation carried forward (LOCF). In case of missing summary statistics data, we will contact the authors of the primary study. When standard errors (SEs) are presented instead of standard deviations (SDs), the former are converted to standard deviations. If both are missing, we estimate SDs from confidence intervals, *t*-values, or *p*-values, as described in the Cochrane Handbook for Systematic Reviews. ^27^ If none of these options is viable, we will contact the study authors. When no information could be obtained, we derive SDs from those of the other studies using a validated imputation technique. ^28^

Odds ratios will be used to calculate dichotomous outcomes. Everyone allocated to the intervention will be counted to determine whether they have completed the follow-up. For beneficial outcomes, patients (e.g., responses) with missing outcome data will be assumed to be non-cases. All effect sizes are presented with 95% confidence intervals (CIs).

The primary time point of interest is 12 months, which, according to Cochrane, can be considered a medium term. The primary outcomes will also be collected and analyzed for up to six months (short-term) and more than 12 months (long-term).

*Risk of bias assessment*

Two reviewers will independently assess the risk of bias of the primary outcomes using the Cochrane Risk of Bias tool, version 2 (RoB 2.0). ^29^ Disagreements between the two reviewers will be discussed with a third senior reviewer (SL, IB). We will not include studies whose sequence generation was judged to show a high risk of bias in the data analyses. The impact of the risk of bias will be analyzed using a sensitivity analysis.

### Data analysis

*Conventional pairwise meta-analyses*

First, we will perform conventional pairwise meta-analyses for specific comparisons covered by the included trials. If the requirements for a network meta-analysis are not met, we present only the findings from pairwise syntheses. Pairwise meta-analyses will be based on a random-effects model with $\tau^{2}$ quantified using the restricted maximum likelihood estimator. ^30^ The confidence intervals of the pooled effect will be adjusted using the method described by Hartung and Knapp. ^31^^,^^32^

*Assessment of heterogeneity*

Heterogeneity will be quantified using the estimated between-study heterogeneity variance ( $\tau^{2}$ ) and will be presented using 95% prediction intervals. In the network meta-analysis model, heterogeneity variance is assumed to be common across the various treatment comparisons, and the empirical distributions are used to characterize the amount of heterogeneity as low, moderate, or high using the first and third quantiles. ^33^^,^^34^ Potential reasons for heterogeneity will be explored using subgroup analysis and meta-regression.

*Network meta-analysis*

Network meta-analysis allows the combination of direct and indirect evidence for all relative treatment effects, and can therefore provide effect estimates with maximum power and increased precision. ^35^ The NMA models will be implemented using the frequentist graph-theoretical framework developed by Rücker. ^36^ NMA models are based on the transitivity assumption, which is checked by investigating whether potential clinical and methodological effect modifiers (see subgroup analysis) are similarly distributed across studies. We will use the design-by-treatment interaction test that evaluates inconsistency from all possible sources in the network jointly, ^37^ as well as the SIDE test (separating indirect evidence from direct evidence), ^38^ assessing the agreement of indirect and direct evidence for every possible comparison in the network. In the case of evidence of inconsistency or intransitivity, we investigated possible sources. Small or moderate amounts of inconsistency will be further explored by network meta-regression and subgroup analyses, using the potential effect modifiers listed below. We estimated the probability for each intervention to be ranked at each possible place, given the relative effect sizes estimated in the NMA. We obtain a hierarchy of competing interventions using a frequentist extension of the surface under the cumulative ranking curve (P-score), as well as the mean ranks. ^39^

*Subgroup analysis and meta-regression*

We plan to conduct the following subgroup analyses and network meta-regressions of the primary outcomes to investigate the impact of potential effect modifiers: a) age, b) patients clearly experiencing a first episode versus broader criteria (e.g., time criteria or second episode), c) duration of illness, d) number of sessions, e) manualized vs. non-manualized interventions, f) dose of antipsychotic treatment, and g) baseline severity of symptoms.

*Sensitivity analyses*

We plan to conduct the following sensitivity analyses of the primary outcomes by excluding: a) studies that did not use a blind outcome assessor (open studies); b) studies presenting only completer analyses; c) overall high risk of bias studies; d) studies with researchers’ allegiance; and e) studies that did not use operationalized diagnostic criteria.

*Small study effects and publication bias*

We will explore the association between study size and effect size using a comparison-adjusted funnel plot method. ^40^ A comparison of more than 10 studies will be plotted in a contour-enhanced funnel plot. Any asymmetry observed can be attributed to systematic differences between small and large studies (e.g., differences in the risk of bias or recruitment), true heterogeneity, or publication bias. If significant small trial effects are identified, we will account for them via network meta-regression using a measure of study uncertainty (e.g., variance) as a covariate. ^40^^,^^41^ The possibility of reporting bias across the entire network will be assessed using the RoB-MEN framework. ^42^^,^^43^

*Evaluating the confidence in estimates*

The confidence in estimates of the main outcomes will be evaluated with the framework Confidence in Network Meta-Analysis (CINeMA), ^44^ an adaptation of the Grading of Recommendations Assessment, Development, and Evaluation framework (GRADE) specifically developed for NMA. The possibility of reporting bias across the entire network will be assessed using the RoB-MEN framework. ^42^ The margin of equivalence for continuous endpoints will be an SMD of 0.2 ^45^ and an OR of 0.8 (1.25) for dichotomous outcomes (cf. Ref. 46).

*Statistical software*

Analyses will be conducted in R. A pairwise meta-analysis will be performed using the “meta” package. ^47^ The frequentist NMA model will be fitted using the “netmeta” package. ^48^ Network meta-regression will be performed in a Bayesian framework using models implemented in JAGS, ^49^ assuming a weakly informative prior for the between-study heterogeneity variance $\tau^{2}$ .

## Discussion

This network meta-analysis will examine the effectiveness of psychological interventions in the treatment early-phase schizophrenia. We will maximize statistical power by combining direct and indirect comparisons and measuring the relative effects and adverse events of different psychological interventions with regard to various outcome measures such as symptomatology, relapses, and quality of life. Based on these findings, it is possible to identify the most effective therapy. Thus, the results have great potential to improve the guidelines and treatment of people with schizophrenia.

Network meta-analysis currently presents the most advanced way to summarize evidence from multiple (in theory, interchangeable) treatments, and the results obtained are highly dependent on the quality of the included studies. We expect different definitions for the first episode and early phase schizophrenia. Considering the variability, we selected broad inclusion criteria regarding the definition to identify a wide range of eligible studies and carefully scrutinize the specific definitions from each study. To investigate the potential resulting heterogeneity, corresponding meta-regression and subgroup analyses will be conducted.

By improving patients’ symptoms and well-being, evidence-based information on efficacious psychological treatments may also reduce the need for medications and the burden of side effects. This evidence-based information is important for the choice of individual treatment plans from a shared decision-making perspective. Our results will show how well various psychological treatments have been studied so far and the limitations of the current evidence base, allowing meaningful planning of future trials. Overall, this should result in a major step forward in terms of individualized treatment of schizophrenia, which can lead to changes in the German and international guidelines.

## Declarations

Ethics approval and consent to participate: not applicable.

Consent for publication: not applicable.

## Authors’ contributions

SL was the principal investigator, obtained funding, and supervised the study. IB and SL designed the study and provided the clinical and methodological advice. LF, MH, IB, and SL drafted the manuscript with input from JP. LF registered the protocol with PROSPERO. LF, NHS, and MH conducted literature screening. WPH contributed to the study from the patient’s perspective. The GS provided substantial methodological and statistical advice. All authors critically reviewed the manuscript for important intellectual content and approved the final version.

## Data Availability

No data are associated with this article. Figshare, AdditionalFile1PRIMA-P-checklist.docx, https://doi.org/10.6084/m9.figshare.25877710.v1 Figshare, Appendix 2_Search Strategy.pdf, https://doi.org/10.6084/m9.figshare.25877710.v1 Data are available under the terms of the Creative Commons Zero “No rights reserved’ data waiver (cc by 4.0). Figshare: Feber, Lena (2024). Supplement to Psychological interventions for early-phase schizophrenia: protocol for a systematic review and network meta-analysis. figshare. Online resource. https://doi.org/10.6084/m9.figshare.25877710.v1. ^50^
