# Supplementary material for: Psychological interventions for early-phase schizophrenia: protocol for a systematic review and network meta-analysis
Source: F1000Res. 2024 Sep 16;13:649. Originally published 2024 Jun 17. [Version 2] doi: 10.12688/f1000research.152191.2 (PMC11375411; doi:10.12688/f1000research.152191.2)
Supplement: Peer review history

# Reviewer report: Reviewer response for version 2

**DOI:** 10.5256/f1000research.171749.r323971

**Published:** 2024-09-18

**Authors:** Stefano Barlati

**Affiliations:** 1 University of Brescia, Brescia, Italy

Authors clearly replied to all the questions raised, modifying the text of the manuscript accordingly.

 Now the manuscript has definitely improved as a whole and could be considered for indexing in its current form.

Is the study design appropriate for the research question?

Yes

Is the rationale for, and objectives of, the study clearly described?

Yes

Are sufficient details of the methods provided to allow replication by others?

Yes

Are the datasets clearly presented in a useable and accessible format?

Yes

Reviewer Expertise:

Schizophrenia; Psychosocial interventions; Cognitive Functioning.

I confirm that I have read this submission and believe that I have an appropriate level of expertise to confirm that it is of an acceptable scientific standard.

# Reviewer report: Reviewer response for version 1

**DOI:** 10.5256/f1000research.166921.r314086

**Published:** 2024-09-04

**Authors:** Joachim Klosterkötter

**Affiliations:** 1 University Hospital Cologne, Cologne, Germany

The authors are interested in two undoubtedly important questions in current therapeutic research:

 Which psychological interventions have already been tested for their potential benefit in the treatment of so-called early-phase schizophrenia?

 Among all the interventions that have been evaluated so far, which one is currently considered the best option for enhancing therapy offerings in the early stages of the illness?

 To scientifically and thoroughly answer these two closely related questions, the authors first plan to compile all randomized controlled trials conducted on such interventions so far through a systematic review. In the second step, they consequently intend to use modern statistical techniques, specifically a network meta-analysis (NMA), to comprehensively compare the effectiveness and tolerability of the various psychotherapies and other psychological or psychosocial treatments used in these clinical studies.

 Their first paper in this context follows the meanwhile well-established practice in therapeutic research of prepublishing the study protocol. This approach provides an opportunity to get acquainted with the intended research project and also allows for suggestions for any necessary improvements before the study begins. This makes a lot of sense, especially for an ambitious and complex project like the planned comparison of all available options in an important therapeutic application field. Because when it comes to evidence syntheses, especially for the development of clinical guidelines, it is crucial to ensure that the proposed strategies for study selection and evaluation analysis truly offer realistic chances of generating new evidence-basing.

 Looking back at conventional meta-analyses, which only ever compared two treatment options with each other, NMAs certainly represent a significant methodological advanced development that is now clearly preferred by all relevant institutions in international treatment research. However, NMAs have also proven to be more prone to misinterpretation than pairwise comparisons, especially when the included studies are too dissimilar, too heterogeneous in their outcomes, or when the directly measured and indirectly derived evidence is too inconsistent. This is precisely why the pre-publication of the study protocol is particularly important for planned "mixed treatment" or "multiple treatment meta-analyses," as NMAs are also known. Only full transparency and critical reflection of the study design within the relevant scientific community can at least to a great extent reliably ensure that in retrospect the application criteria for NMAs had not been met.

 However, the planned NMA would not be the first to focus on psychological interventions for patients with schizophrenia. The authors are well aware that there have already been three such intervention comparisons using the new network method. However, they argue that the studies included in these previous NMAs either focused only on certain aspects of symptomatology [1] and disease progression [2] or were conducted in the distant past [3]. In contrast, their systematic review and new NMA, which they announce with the pre-publication of this study protocol, are designed to be much more comprehensive and up-to-date in terms of the planned study selection.

 Indeed, the protocol communications regarding the eligibility criteria and the primary and secondary outcomes establish an extraordinarily broad scope for data collection. All psychological interventions that meet the classification criteria proposed by the Cochrane Common Mental Disorders Group (formerly CCDAN) are to be considered if they have been investigated in randomized controlled trials. The authors expect that various forms of therapy, including art and music therapy, social skills training, and family interventions, were administered in the majority of studies in addition to standard care, which usually included antipsychotic medication. This further strongly emphasizes that their analysis genuinely aims to improve the evidence base for current clinical practices.

 All specifications about the methods, including those related to the search strategy, the identification of selection of studies, and data extraction, as well as those concerning the most important aspect of the planned investigation, namely data analysis, appear plausible. If all these steps can be implemented as planned, it should indeed be possible to establish a ranking from the best to the worst psychological interventions. However, a potential issue is also emerging here that cannot yet be determined before the systematic review has been carried out as to whether it could significantly jeopardize the applicability of the planned NMA.

 The studies approved under the protocol are supposed to focus on participants aged over 16 and under 65 years with a recent first-time diagnosis of psychosis, first episode schizophrenia, or early-phase schizophrenia. The authors intend to define the somewhat unclear terms "early-phase schizophrenia" or "recent onset psychosis" by a maximum illness duration of 5 years. All participants in the approved studies must present or have presented full-blown psychotic symptoms. For this reason, studies on populations at risk of psychosis or in the prodrome phase are excluded, even though the psychological interventions of interest here actually play an even greater role as recognized first-choice prevention strategies in those populations [4]. Ultimately, all studies relating to manifest schizophrenic, schizoaffective, and delusional disorders with a duration of up to 5 years are included, regardless of how many psychotic episodes have occurred within that period. However, this is a very broad inclusion range, for which the title "early-phase schizophrenia" might seem misleading. The authors themselves are concerned about a potential resulting heterogeneity that could jeopardize the requirements for comparability of all included studies that apply to the NMAs. Therefore, it would be helpful if they could elaborate further on their suggested methods for capturing and managing such heterogeneity and how they intend to deal with it.

 However, from my perspective, this is the only minor improvement suggestion for a study protocol that otherwise indeed promises to be a major step forward in terms of individualized treatment for schizophrenia

Is the study design appropriate for the research question?

Yes

Is the rationale for, and objectives of, the study clearly described?

Yes

Are sufficient details of the methods provided to allow replication by others?

Yes

Are the datasets clearly presented in a useable and accessible format?

Yes

Reviewer Expertise:

Early recognition and intervention of psychoses. Prevention of mental disorders.

I confirm that I have read this submission and believe that I have an appropriate level of expertise to confirm that it is of an acceptable scientific standard.

## References

[1] : Psychological interventions to reduce positive symptoms in schizophrenia: systematic review and network meta-analysis. *World Psychiatry* .2018;17(3) : 10.1002/wps.20577 316-329 10.1002/wps.20577 30192101 PMC6127754

[2] : Psychosocial and psychological interventions for relapse prevention in schizophrenia: a systematic review and network meta-analysis. *The Lancet Psychiatry* .2021;8(11) : 10.1016/S2215-0366(21)00243-1 969-980 10.1016/S2215-0366(21)00243-1 34653393

[3] : A network meta-analysis of psychological interventions for schizophrenia and psychosis: Impact on symptoms. *Schizophrenia Research* .2021;228: 10.1016/j.schres.2020.12.036 447-459 10.1016/j.schres.2020.12.036 33578368

[4] : Prevention of First-Episode Psychosis in People at Clinical High Risk: A Randomized Controlled, Multicentre Trial Comparing Cognitive-Behavioral Therapy and Clinical Management Plus Low-Dose Aripiprazole or Placebo (PREVENT). *Schizophr Bull* .2023;49(4) : 10.1093/schbul/sbad029 1055-1066 10.1093/schbul/sbad029 37021666 PMC10318879

# Reviewer report: Reviewer response for version 1

**DOI:** 10.5256/f1000research.166921.r314091

**Published:** 2024-08-19

**Authors:** Stefano Barlati

**Affiliations:** 1 University of Brescia, Brescia, Italy

1) SUMMARY OF ANALYZED ARTICLE

 The present study protocol for a systematic review and network meta-analysis regarding psychological interventions for early-phase schizophrenia aims to develop a hierarchy of existing psychological treatments concerning their efficacy and tolerability, which will inform treatment guidelines.

 2) STRUCTURE

 The protocol has all the key elements: abstract, introduction, methodology, data analysis, and discussion.

 a) Title

 The title clearly describes the manuscript.

 b) Abstract

 The abstract is well composed, it is clearly wrote and it well reflects the content of the article.

 c) Introduction

 The introduction describes what authors hoped to achieve and it explains relevant previous researches on this topic.

 d) Methodology

 Authors explain materials and methods. In particular they well describe the study design, eligibility criteria, search strategy, outcomes, and statistical analysis.

 e) Discussion /Conclusions

 Authors indicate how the present protocol / study could contain useful information, not only in a research perspective, but also in clinical practice. This is a topic of interest for growing literature and authors explain how this research can be informative for future studies.

 g) Figures and tables

 The supplementary figures well inform the reader and they are consistent for the aim of this paper.

 3) LANGUAGE

 The article is written in correct and fluent English.

 4) RECOMMENDATION

 The protocol / manuscript may have a significant interest and bring new perspectives for research in provide useful evidence-based information for the choice of individual treatment plans from a shared decision-making perspective.

Is the study design appropriate for the research question?

Yes

Is the rationale for, and objectives of, the study clearly described?

Yes

Are sufficient details of the methods provided to allow replication by others?

Yes

Are the datasets clearly presented in a useable and accessible format?

Yes

Reviewer Expertise:

I'm involved in research projects on clinical, biological, pharmacological and rehabilitative aspects of mental disorders, in particular schizophrenia, mood disorders, autism and personality disorders.

I confirm that I have read this submission and believe that I have an appropriate level of expertise to confirm that it is of an acceptable scientific standard.

# Reviewer report: Reviewer response for version 1

**DOI:** 10.5256/f1000research.166921.r303417

**Published:** 2024-08-01

**Authors:** Alexandre Hudon

**Affiliations:** 1 University of Montreal, Montreal, Québec, Canada

Brief summary: The authors of this interesting protocol provide a methodology to conduct a systematic review and network meta-analysis of psychological interventions for early-phase schizophrenia. Please see my comments below.

**Abstract:**

 - The introduction segment is very broad, almost as-if AI generated. Contextualising better the introduction by providing specific elements of early phase schizophrenia could be beneficial for the readership.

 - The protocol section is vague. Considering this is a systematic review, it was thought that details about the search strategy would be provided.

**Introduction:**

 - The word debilitating could be offending to certain patients. The authors are encouraged to use another term. 

 - Considering the main concept is early phase schizophrenia, the main prodromes of schizophrenia should be briefly discussed (e.g. vunerability group, attenuated psychosis group and BLIPS group which are the groups with the most evidence up to this day) as there is relevance regarding the therapeutic interventions for these groups.

 - Please clarify the aim of this review as well as the main hypothesis.

 Overall the introduction is well written with relevant literature.

**Eligibility criteria:**

 - Main eligibility criteria should include specific criteria relevant to early phase schizophrenia considering the topic of this review.

 - What are the exclusion criteria?

**Secondary outcomes:**

 - Please provide psychometric properties and rationale for the scales used.

 For the remaining portions of this protocol I have no further comment as it is clearly written and easy to follow.

Is the study design appropriate for the research question?

Yes

Is the rationale for, and objectives of, the study clearly described?

Yes

Are sufficient details of the methods provided to allow replication by others?

Yes

Are the datasets clearly presented in a useable and accessible format?

Not applicable

Reviewer Expertise:

Schizophrenia, Mental Health, Psychiatry, Virtual reality, Artificial Intelligence

I confirm that I have read this submission and believe that I have an appropriate level of expertise to confirm that it is of an acceptable scientific standard, however I have significant reservations, as outlined above.

# Response

**Published:** 2024-09-10

**Authors:** Lena Feber

**Affiliations:** Psychiatry and Psychotherapy, Technical University of Munich, TUM School of Medicine and Health, Klinikum rechts der Isar, Department of Psychiatry and Psychotherapy, Munich, Germany, Munich, Germany

**Abstract:**

 The introduction segment is very broad, almost as-if AI generated. Contextualising better the introduction by providing specific elements of early phase schizophrenia could be beneficial for the readership.

**Answer: Thank you very much for your comment. We can certify that none of the contents of this paper have been written with the use of LLMs or other AI-based tools. We also changed the text for a better focus on early phase (abstract): “Pharmacotherapies are first-line treatments, but have their own limitations. There is a consensus on the need of non-pharmacological interventions for individuals in the early phase of schizophrenia. The early phase refers to the onset of the illness, often also to the point at which the symptoms first appeared or were diagnosed.”**

 The protocol section is vague. Considering this is a systematic review, it was thought that details about the search strategy would be provided.

**Answer: Thank you very much for pointing out that additional information is needed here. We have now added (abstract): "We will search multiple electronic databases and additionally, statements of guidelines and specific relevant journals concerning psychological treatments for schizophrenia in order to retrieve other studies. The search strategy will combine terms for psychological interventions, early-phase schizophrenia and randomization.”**

**Introduction:**

 The word debilitating could be offending to certain patients. The authors are encouraged to use another term. 

**Answer: Thank you for this important feedback, we have now deleted the word and replaced it with the word “severe”.**

**Adapted (introduction): “Schizophrenia is a debilitating and severe and often life-long disorder, ranking among the 20 top causes of disability (1).”**

 Considering the main concept is early phase schizophrenia, the main prodromes of schizophrenia should be briefly discussed (e.g. vunerability group, attenuated psychosis group and BLIPS group which are the groups with the most evidence up to this day) as there is relevance regarding the therapeutic interventions for these groups.

**Answer: Thank you for your important comment. We also acknowledge that these essential groups can be associated with the development of schizophrenia. However, we intentionally choose not to include these groups in our review since our criterion is that a diagnosis already exists. It is possible that within the at-risk groups, ultimately, no illness or a different illness may develop.**

**Already in the text (characteristics of participants):**

**We will exclude studies in “at risk of psychosis” or “prodrome” populations, because such patients do not experience full-blown psychotic symptoms yet by definition.**

**Added tot he introduction:**

**„Schizophrenia can also manifest in the prelude to the full onset of symptoms through early warning signs such as social withdrawal, mistrust, sleep disturbances, and many more 6. However, these are not definitive indicators of the disorder but can be viewed as early warning signs.”**

**We also clarified in the section “characteristics of patients” now: “Participants must exhibit symptoms that match the criteria for schizophrenia or related disorders (schizophreniform, schizoaffective or delusional disorder, however diagnosed). Individuals with a specific vulnerability, brief limited intermittent psychotic symptoms or light psychotic symptoms will be excluded since the symptoms do not definitively suggest the development of schizophrenia.”**

 Please clarify the aim of this review as well as the main hypothesis.

**Answer: Thank you for the suggestion to address this more clearly. We have made some additions.**

**Already in the text:**

**“Knowledge of psychological interventions that are most efficacious in improving symptoms and preventing relapse in this critical population may allow to improve treatment planning and resource allocation in the health care sector.”**

**“Thus, it is currently unclear which are the most efficacious and acceptable psychological treatments for patients with schizophrenia at the early phase of the disorder. This underlines the need for a network meta-analysis producing a comprehensive synthesis of all the randomised evidence focusing on this particular population.”**

**Added (objective):**

**“Finding out which psychological intervention is most efficacious in treating patients with schizophrenia in the early phase can have a decisive impact on preventing chronicity of the illness. The results of this work may hold significant relevance for resource planning in treatment.”**

 Overall the introduction is well written with relevant literature.

**Eligibility criteria:**

 Main eligibility criteria should include specific criteria relevant to early phase schizophrenia considering the topic of this review.

**Answer: Thank you for your important comment. Our inclusion criteria relevant to early phase schizophrenia are the following:**

**- “We will consider trials in patients with a recent first-time diagnosis of psychosis, first-episode schizophrenia or early-phase schizophrenia, as per the inclusion criteria of the specific studies.” (patient characteristics)**

**–“In cases of “early-phase schizophrenia” or “recent-onset psychosis” we will accept a maximum duration of illness of 5 years.” (patient characteristics)**

**-“We will also include studies conducted in specialized early intervention centers, since they target the population in question.” (patient characteristics)**

**-“We will include studies with participants who are aged 16 years or older, allowing to capture the variability in age of onset of schizophrenia.” (patient characteristics)**

 What are the exclusion criteria?

**Answer: Thank you for your inquiry. We have mentioned the respective exclusion criteria in the specific sections (characteristics of studies, characteristics of participants, interventions):**

**-“We will exclude studies which only included patients with bipolar mania, bipolar depression, substance-induced disorders, and psychoses of organic origin.” (patient characteristics)**

**-“We will exclude studies in “at risk of psychosis” or “prodrome” populations, because such patients do not experience full-blown psychotic symptoms yet by definition.” (patient characteristics)**

**-“Organisation of care-oriented interventions (e.g. case management, assertive community treatment) and supported employment will not be considered, because they have other aims, and it will not be meaningful to analyse their efficacy in contrast to the ones mentioned above.”(interventions)**

**Secondary outcomes:**

 Please provide psychometric properties and rationale for the scales used.

**Answer: Thank you for the feedback that additional information regarding the scales for the secondary outcomes is needed. As already specified we will accept any validated scale that is used in the original studies.**

**We added (secondary outcomes): “We will focus on validated and established scales. Other rating scales will only be used if the instrument has been published in a peer-reviewed journal, because non-validated schizophrenia scales tend to exaggerate differences 22.”**

 For the remaining portions of this protocol I have no further comment as it is clearly written and easy to follow.
